# How Will We React to the Discovery of Extraterrestrial Life?

**DOI:** 10.3389/fpsyg.2017.02308

**Published:** 2018-01-10

**Authors:** Jung Yul Kwon, Hannah L. Bercovici, Katja Cunningham, Michael E. W. Varnum

**Affiliations:** ^1^Department of Psychology, Arizona State University, Tempe, AZ, United States; ^2^School of Earth and Space Exploration, Arizona State University, Tempe, AZ, United States; ^3^Interplanetary Initiative, Arizona State University, Tempe, AZ, United States

**Keywords:** extraterrestrial life, societal reactions, LIWC, affect, scientific discovery

## Abstract

How will humanity react to the discovery of extraterrestrial life? Speculation on this topic abounds, but empirical research is practically non-existent. We report the results of three empirical studies assessing psychological reactions to the discovery of extraterrestrial life using the Linguistic Inquiry and Word Count (LIWC) text analysis software. We examined language use in media coverage of past discovery announcements of this nature, with a focus on extraterrestrial microbial life (Pilot Study). A large online sample (*N* = 501) was asked to write about their own and humanity’s reaction to a hypothetical announcement of such a discovery (Study 1), and an independent, large online sample (*N* = 256) was asked to read and respond to a newspaper story about the claim that fossilized extraterrestrial microbial life had been found in a meteorite of Martian origin (Study 2). Across these studies, we found that reactions were significantly more positive than negative, and more reward vs. risk oriented. A mini-meta-analysis revealed large overall effect sizes (positive vs. negative affect language: *g* = 0.98; reward vs. risk language: *g* = 0.81). We also found that people’s forecasts of their own reactions showed a greater positivity bias than their forecasts of humanity’s reactions (Study 1), and that responses to reading an actual announcement of the discovery of extraterrestrial microbial life showed a greater positivity bias than responses to reading an actual announcement of the creation of man-made synthetic life (Study 2). Taken together, this work suggests that our reactions to a future confirmed discovery of microbial extraterrestrial life are likely to be fairly positive.

## Introduction

How will we react to the discovery of alien life? In 1953, the Robertson Panel warned of the danger of mass hysteria (Durant, 1953), and a recent national poll found that 25% of American respondents anticipated people would panic (Harrison, 2011). Depictions of contact with extraterrestrial life in fiction for over a century have highlighted potential downsides of alien contact, from H. G. Wells’ “War of the Worlds” (Wells, 1898/2003), to the television series “The X-Files” (Carter, 1993–2002), and films such as “The Day the Earth Stood Still” (Blaustein and Wise, 1951), “Independence Day” (Devlin and Emmerich, 1996), and “Edge of Tomorrow” (Hoffs et al., 2014). However, most speculations regarding humanity’s reactions to extraterrestrial life, both in fiction and otherwise, have focused on discovering evidence of intelligent life from elsewhere, while less consideration has been given to how we may react to the discovery of extraterrestrial life that is not intelligent, even though we are more likely to encounter microbial life in our solar system (Race and Randolph, 2002; Race, 2008; Gronstal, 2013). Some scientists, including Ramin Skibba, have suggested that the discovery of any extraterrestrial life, even in microbial forms, may be “earth-shattering” (Skibba, 2017). Other experts, including scientists such as Christof Koch, Guy Consolmagno, and Aaron Gronstal, have suggested that the discovery of extraterrestrial microbial life will have little in the way of societal or psychological impact (Gronstal, 2013; Levine, 2016). To date, though, the only empirical work of which we are aware that assessed potential psychological reactions to extraterrestrial life has done so by positing hypothetical contact with an intelligent extraterrestrial species (Vakoch and Lee, 2000).

Thus, although the question of how we will react to extraterrestrial microbial life has spawned much speculation, it has sparked scant empirical work, and none that we are aware of which addressed reactions to actual announcements of such a discovery. In the present series of studies, we sought to provide an initial, yet systematic, test of psychological reactions to the discovery of extraterrestrial life. To do so, we conducted quantitative analyses of media coverage of past reactions to announcements of this nature (Pilot Study); individuals’ predictions regarding their own reactions, and those of humanity as a whole, to a hypothetical discovery of extraterrestrial life (Study 1); and, lastly, individuals’ reactions to media coverage of a past announcement of the discovery of evidence that suggested there was once life on Mars (Study 2). In these studies, we focused on reactions to extraterrestrial microbial life, as opposed to intelligent life, as the Drake Equation^1^, suggests it is far more probable that we discover evidence of this type of life, considering direct exploration of our solar system has so far ruled out the possibility that we share it with intelligent extraterrestrial beings. Potential remains for the discovery of microbial life in our solar system, which is why extraterrestrial microbes are the focus of our study.

In the present set of studies we focused on affective reactions (positive vs. negative) to discovery of extraterrestrial microbial life, as well as whether announcements of such discoveries, or the prospect of them, produced a greater orientation to reward vs. risk. To do so, we primarily conducted quantitative analyses of natural language use in response to such discoveries, a method that has been used to assess affective states, drives, personality, and mental health in a large body of prior research (for a review, see Pennebaker et al., 2003). More recently, this approach has been used to assess a variety of novel questions including the affective states of people facing death (Hirschmüller and Egloff, 2016; Goranson et al., 2017), and cultural shifts in gender equality (Varnum and Grossmann, 2016). In the present work, we used the Linguistic Inquiry and Word Count (LIWC; Pennebaker et al., 2015) text analysis software to analyze media accounts, government statements, and press releases regarding discoveries potentially indicative of extraterrestrial life, with a particular focus on the 1996 announcement of evidence for extraterrestrial microbial life (Pilot Study). We generated predictions for Studies 1 and 2 based on the results of this pilot study, and proceeded to assess affective and risk vs. reward oriented reactions to a hypothetical announcement of the discovery of extraterrestrial microbial life (Study 1), as well as reactions to media coverage of the 1996 announcement (Study 2), as a way to assess people’s actual reactions to such information.

## Pilot Study: Media Coverage of Discovery of Extraterrestrial Microbial Life

In a pilot study, we sought to provide an initial assessment of past societal responses to announcements of the discovery of extraterrestrial life, or discoveries that might suggest this possibility. Analysis of language in news coverage and other cultural products has been used in a number of previous studies to assess affective states, values, and attitudes at the cultural level (e.g., Greenfield, 2013; Grossmann and Varnum, 2015; Iliev et al., 2016; Varnum and Grossmann, 2016), as well as at the individual level (e.g., Danner et al., 2001; Pennebaker et al., 2003; Goranson et al., 2017). We analyzed the language used in past news articles about discoveries of evidence for extraterrestrial life to examine whether such events are portrayed in a generally positive or negative light.

### Method

We identified five relevant discovery events: (1) the 1967 discovery of pulsars which were initially thought to be potential extraterrestrial broadcasts, (2) the 1977 Wow signal, which was also thought to be potential extraterrestrial broadcasts, (3) the 1996 discovery of potential fossilized extraterrestrial microbes in a meteorite of Martian origin, (4) the 2015 discovery of periodic dimming around Tabby’s Star which was thought to potentially indicate the presence of an artificially constructed Dyson sphere around the star, and (5) the 2017 discovery of numerous Earth-like exoplanets in the habitable zone of a star. Fifteen news articles providing contemporaneous media coverage of three of the above events suggesting evidence for extraterrestrial life were selected from various publications, including the New York Times, the Wall Street Journal, the Washington Post, Time Magazine, and Science Magazine. We also included any contemporaneous announcements made by NASA or the Federal Government, and, in the case of Tabby’s Star, coverage from the Atlantic.com and Space.com. For Tabby’s Star we could not find any coverage in our pre-specified list but hoped to include the event in order to explore the nature of affective reactions to a variety of discoveries that might be suggestive of different types of extraterrestrial life. We thus used news coverage from the first two sources that appeared to be of high journalistic quality. Seven of the articles were about the discovery of evidence for microbial life from a Martian meteorite in 1996, two articles were about the discovery of a potential Dyson sphere around Tabby’s Star in 2015, and six articles were about NASA’s discovery of Earth-like exoplanets in 2017.

The LIWC software (Pennebaker et al., 2015) was used to determine what percentage of the total words in each article reflected positive affect, negative affect, reward, or risk. Words were categorized according to the default LIWC2015 dictionary. LIWC calculates the percentages of words in a text which reflect various psychological states, feelings, or parts of speech. Typically, these values are small and LIWC’s standard output reports 1% as 1.00, 0.1% as 0.10, etc. Thus values reported throughout this manuscript are based on percentages. This practice is standard in other articles reporting LIWC results (i.e., Hirschmüller and Egloff, 2016) and we follow it order to make it easier to compare our results with other published work using LIWC.

### Results

Linguistic Inquiry and Word Count text analyses of all 15 articles together and subsequent paired-samples *t*-tests revealed that words describing positive affect (*M* = 1.33, *SD* = 0.49) were more prevalent than those describing negative affect (*M* = 0.50, *SD* = 0.48), *t*(14) = 6.01, *p* < 0.001, *d* = 1.71. Words reflecting reward orientation (*M* = 0.44, *SD* = 0.21) appeared more frequently than those reflecting risk orientation (*M* = 0.12, *SD* = 0.11), *t*(14) = 5.56, *p* < 0.001, *d* = 1.90.

We also examined whether these results might differ across the three events, as they are indicative of non-intelligent life (microbial life on Mars), intelligent life (Dyson sphere around Tabby’s Star), or life in other Earth-like exoplanets which may or may not be intelligent. A two-way mixed-design ANOVA revealed no interaction between event (Mars meteorite vs. Tabby’s Star vs. System of Earth-like Planets) and affect (positive vs. negative), *F*(2,12) = 0.63, *p* = 0.55, $\eta_{p}^{2}$ = 0.095. However, there was a significant interaction between event and reward vs. risk, *F*(2,12) = 6.70, *p* = 0.011, $\eta_{p}^{2}$ = 0.527. *Post hoc* Tukey comparisons showed that the difference between the percentages of words reflecting reward and words reflecting risk was significantly larger for the articles about possibility of life on Earth-like exoplanets (*M* = 0.50, *SD* = 0.20) than for the articles about microbial life on Mars (*M* = 0.17, *SD* = 0.14) at *p* = 0.009.

**FIGURE 1:**
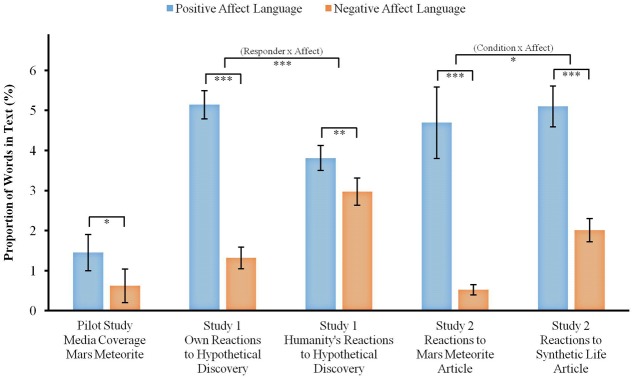
Differences in the percentage of words reflecting positive vs. negative affect in reactions to the discovery of Martian microbial life in each study. Error bars indicate 95% confidence intervals. ^∗^*p* ≤ 0.05, ^∗∗^*p* = 0.001, ^∗∗∗^*p* < 0.001.

As it is most likely that we will first discover extraterrestrial life in the form of microbes, in a separate set of analyses, we focused on coverage of the seven articles from 1996 about the evidence of life from a Martian meteorite. We found similar results, indicating that these articles also contained more words reflecting positive affect (*M* = 1.45, *SD* = 0.61) compared to those reflecting negative affect (*M* = 0.62, *SD* = 0.56), *t*(6) = 3.34, *p* = 0.016, *d* = 1.40 (see **Figure 1**), as well as more words reflecting reward (*M* = 0.32, *SD* = 0.15) compared to those reflecting risk (*M* = 0.16, *SD* = 0.13), *t*(6) = 3.11, *p* = 0.021, *d* = 1.18.

### Discussion

Results of the Pilot Study suggest that reactions to past announcements of extraterrestrial life discovery (or evidence that suggests such life may exist) are largely positive, indicating greater positive vs. negative affect and more emphasis on potential rewards vs. risks. To the extent that media coverage reflects the broader cultural mood, these findings suggest that society is likely to react in a positive fashion if we were to discover extraterrestrial life in the future. In our two main studies, we sought to test whether individual reactions might also show this pattern in response to the discovery of extraterrestrial microbial life.

## Study 1: Predicted Reactions to the Discovery of Extraterrestrial Microbial Life

Given that it is more likely we will discover evidence of microbial extraterrestrial life than intelligent extraterrestrial civilizations, in Studies 1 and 2 we assessed reactions to the discovery of extraterrestrial microbes. In Study 1, we assessed people’s beliefs regarding how both they and humanity as a whole might react to such a discovery. To do so, we asked participants to imagine a scenario in which such an announcement was made and to describe how they would react in a free response format. As an exploratory question, we also asked whether individuals’ forecasts of their reactions might differ from their forecasts for how humanity as a whole would react. Participants were thus asked to describe how humanity would react to the same announcement.

### Preregistered Predictions

Before data collection, we preregistered predictions, the full materials we planned to use in the study, the target sample size (*N* = 500), and rules regarding data exclusion, on 9/6/2017 for Study 1 at the Open Science Framework (OSF, osf.io/mgkau). We collected data online using subjects from Amazon MTurk on 9/13/2017.

Based on results from the Pilot study, in Study 1, we predicted that participants’ written responses to a hypothetical discovery of extraterrestrial microbial life would reflect more positive vs. negative affect, and more reward vs. risk orientation. We also predicted that their scores on a modified version of the Positive and Negative Affect Schedule (PANAS; Watson et al., 1988) in response to this hypothetical discovery would be greater for the positive scale than negative scale, and that responses to the two close-ended items regarding potential rewards vs. risks of such a discovery would show greater perceived potential rewards than risks (for materials, see osf.io/mgkau). We did not make predictions regarding potential interactions between condition (own reaction vs. humanity’s) and affect or condition and reward vs. risk, although we noted in our preregistered predictions that we would assess these potential interactions.

### Method

#### Participants

Participants (*N* = 504) recruited from Amazon Mechanical Turk (247 females, 4 preferred not to answer; 393 White/European–American, 34 Asian–American, 31 African–American, 27 Latino/Latina–American, 17 other, 2 did not answer) took part in the study. Mean age was 36.3 (*SD* = 10.84), ranging from 18 to 70. Median household income category was $25,000 to $49,999. The most frequent level of education was 4-year college degree (39.3%), followed by some college or 2-year college degree (37.5%), high school diploma (11.3%), and graduate degree (10.9%). Participants also rated their political orientation on a 7-point Likert scale, with 50.6% falling on the liberal side of the scale, 19.3% on the midpoint (moderate), and 30% on the conservative side. Participants were paid $1.00 to complete the survey (mean completion time = 7′ 36′′, *SD* = 3′ 52′′). In order to be eligible to participate, participants had to be located in the United States and have a lifetime HIT approval rate of 95% or higher. Although we ceased data collection upon receiving notification of completion from the target sample size (*N* = 500), the final sample size was slightly greater, as we included all open format responses and fully completed instruments regardless of whether participants skipped items, discontinued participation, or failed to submit their HITs immediately after participation^2^. Inclusion criteria for each analysis are as follows. Participants who provided a random sequence of characters, or failed to respond, to an open response question were excluded from the corresponding text analysis. Those who fully completed the Likert-scale measurements of reactions were included in the analyses even if they did not provide responses to the open format questions. Two participants were excluded from both text analyses (own reactions vs. humanity’s reactions) because they provided a random sequence of letters or a blank for both prompts. For each prompt, there was a participant who responded to only one of the prompts. This resulted in three participants being excluded from each text analysis, leaving *N* = 501 for the paired-samples *t*-tests. Pairwise deletion was used for correlation analyses, resulting in *N*’s ranging from 490 to 501.

#### Procedure

After providing informed consent, participants were asked to imagine that scientists had just announced the discovery of microbial life outside of Earth. They were then asked to think about how they would react to such an announcement, and describe their reactions in an open response format. Participants were also asked to describe how humanity would react to the same kind of announcement. These two tasks (own reaction vs. humanity’s reaction) were presented in random order. For the own reaction condition, the prompt read, “Please take a moment to imagine that scientists have just announced the discovery of the existence of microbial life (i.e., bacteria, viruses, or other similar life forms) outside of planet Earth. Think about how YOU personally would react to such news and please describe how YOU would react below. Please provide as much detail as you can and please try to write at least a few sentences describing what YOUR thoughts, feelings, and responses would be.” The prompt was identical for the humanity’s reaction condition, with the second person pronouns replaced with the phrase “humanity”. Participants then completed a modified version of the PANAS (Watson et al., 1988) that consisted of the first 10-items of the scale (α = 0.74 for the positive affect subscale, and (α = 0.92 for the negative affect subscale; See osf.io/mgkau for scale items), and instructions modified so that participants were instructed to indicate to what extent they would feel these 10 emotions if they “learned that microbial life had been discovered outside of planet Earth” (see osf.io/mgkau for copies of full materials used in this study and Study 2). Participants were also asked to indicate the degree to which the statements, “I would be concerned about potential risks” and “I would be excited about potential opportunities and rewards”, described their reactions using a 7-point Likert scale (1 strongly agree, 7 strongly disagree). Participants also completed the Ten -Item Personality Inventory (TIPI; Openness: (α = 0.52, Conscientiousness: α = 0.67, Extraversion: α = 0.80, Agreeableness: α = 0.50; Emotional Stability: α = 0.78; Gosling et al., 2003), the 6-item Disease Avoidance subscale of the Fundamental Social Motives Inventory (α = 0.91; Neel et al., 2016), and demographic questions including items assessing age, gender, ethnicity, country of residence, country of birth, income, education, and political orientation (see osf.io/mgkau). Study 1 was approved by the institutional review board at Arizona State University.

### Results

#### Participants’ Own Reactions

Linguistic Inquiry and Word Count analysis followed by paired-samples *t*-tests revealed that participants used more words reflecting positive (*M* = 5.14, *SD* = 4.03) than negative affect (*M* = 1.32, *SD* = 3.06) when describing their own hypothetical reactions to the discovery of extraterrestrial microbial life, *t*(500) = 16.91, *p* < 0.001, *d* = 1.07 (see **Figure 1**). Analysis of the PANAS scores showed that participants reported they would feel more positive (*M* = 15.68, *SD* = 4.81) than negative emotions (*M* = 8.83, *SD* = 5.04) in response to such announcement, *t*(489) = 22.44, *p* < 0.001, *d* = 1.39. Participants also used more words reflecting reward (*M* = 1.89, *SD* = 2.59) than risk (*M* = 0.30, *SD* = 1.08), *t*(500) = 12.53, *p* < 0.001, *d* = 0.80. However, contrary to our predictions, responses to the Likert-scale items assessing perceived potential risks and rewards of such a discovery indicated that participants perceived the hypothetical discovery as presenting greater risks (*M* = 4.00, *SD* = 1.96) than rewards (*M* = 2.52, *SD* = 1.66), *t*(502) = 13.15, *p* < 0.001, *d* = 0.82.

#### Humanity’s Reactions

When asked to describe how humanity would react to the same announcement, participants used more words reflecting positive (*M* = 3.81, *SD* = 3.49) than negative affect (*M* = 2.97, *SD* = 3.92), *t*(500) = 3.21, *p* = 0.001, *d* = 0.23 (see **Figure 1**), and more words reflecting reward (*M* = 1.52, *SD* = 2.21) than risk (*M* = 0.46, *SD* = 1.37), *t*(500) = 9.00, *p* < 0.001, *d* = 0.57.

A two-way repeated-measures ANOVA with language affect (positive vs. negative) and responder to the announcement (own vs. humanity) found a significant interaction, *F*(1,499) = 87.08, *p* < 0.001, $\eta_{p}^{2}$ = 0.15. A significant interaction was also found with reward vs. risk and own reaction vs. humanity’s reaction, *F*(1,499) = 10.74, *p* = 0.001, $\eta_{p}^{2}$ = 0.021. These results indicate that the mean differences between the proportions of words reflecting positive vs. negative affect and reward vs. risk were larger for participants’ own reactions compared to their description of humanity’s reactions to the hypothetical discovery of extraterrestrial microbial life.

#### Individual Differences

We did not find particularly strong or consistent correlations between our dependent variables and our individual difference and demographic measures. Given the large number of variables measured, we report here only correlations with an absolute value of 0.2 or above. We observed one such correlation, a positive correlation between self-reported disease avoidance motive and the Likert-scale measure of risk orientation, *r*(498) = 0.21, *p* < 0.001. Full correlation matrices, including correlations among dependent variables can be found at osf.io/mgkau and are also available in the **Appendix**.

### Discussion

Our results were largely consistent with the pattern observed in the Pilot Study. People believe that they will react positively to the discovery of extraterrestrial microbial life and that humanity as a whole will do the same. The only exception to this pattern, and the only finding that contradicted our preregistered predictions was the finding from the two close-ended Likert-scale items assessing potential reward and risk, where people indicated that they would perceive more risk than reward. We do not attempt a strong interpretation of this discrepancy, although we offer some suggestions and future directions based on it in the general discussion.

Interestingly, people anticipate that their own reactions would be more positive than those of humanity as a whole. This may suggest some element of illusory superiority in people’s forecasts regarding reactions to a discovery of extraterrestrial life. However, as we did not address perceived social desirability of different responses to such an event, this remains a question for future research (see section “General Discussion”). In summary, results of this study suggest that people believe, on the whole, both themselves and humanity will respond in positive ways if a confirmed discovery of extraterrestrial microbial life is made.

## Study 2: Actual Reactions to the Discovery of Extraterrestrial Life

In Study 2, we investigated whether the same effects would be observed when people read and responded to an *actual* past announcement of the discovery of extraterrestrial microbial life. Given previous work suggesting that people are not particularly accurate at affective forecasting (e.g., Gilbert et al., 1998; Gilbert and Ebert, 2002; Kushlev and Dunn, 2012), it may be the case that people’s beliefs regarding how they would feel when confronted with such news may not be good predictors of how they would actually react. Thus, in Study 2 we presented an independent sample with a New York Times article from 1996 describing the announcement of fossilized extraterrestrial microbes in a Martian meteorite, in order to assess whether a similar positivity bias might emerge as observed when people were asked to imagine their responses (or humanity’s) to such a discovery (Study 1), or as observed in contemporaneous media coverage of that discovery (Pilot). We also wanted to test whether the positivity bias observed in Study 1 was perhaps unique to the discovery of extraterrestrial life, as opposed to scientific discoveries in general, or to the creation of anthropogenic life. To do so, we conducted a between-subjects experiment in which participants were randomly assigned to read one of two New York Times articles describing either the 1996 Mars meteorite extraterrestrial microbial life announcement or the 2010 announcement of the creation of life by Craig Venter’s lab.

### Preregistered Predictions

Before data collection we preregistered predictions, the full materials we planned to use in the study, the target sample size (*N* = 500), and rules regarding data exclusion on 9/6/2017 for this study on OSF (osf.io/mgkau). We collected data online using an independent sample of subjects from Amazon MTurk on 9/13/2017.

Based on results from the Pilot study, in Study 2, we predicted that participants’ written responses to the discovery of extraterrestrial microbial life would reflect more positive vs. negative affect and more reward vs. risk orientation. We also predicted that PANAS scores in response to this hypothetical discovery would be more positive than negative. However, due to a programming error, a PANAS scale was not included in the experiment (for more details see osf.io/mgkau). We did not make predictions regarding potential interactions between condition and affect, or condition and reward vs. risk, although we noted in our preregistered predictions that we would assess these potential interactions.

### Method

#### Participants

Participants (*N* = 508) recruited from Amazon Mechanical Turk (246 females, 5 preferred not to answer; 381 White/European–American, 42 African–American, 39 Asian–American, 27 Latino/Latina–American, 17 other, 2 did not answer) took part in the study. Mean age was 37.1 (*SD* = 11.63), ranging from 18 to 73. Median household income category was $25,000 to $49,999. Forty percent of participants held a 4-year college degree, followed by some college or 2-year college degree (33.7%), graduate degree (15.2%), and high school diploma (10.4%). Participants also rated their political orientation on a 7-point Likert scale, with 49.7% self-identified as liberal, 24.8% as moderate, and 25.4% as conservative. Participants were paid $1.00 to complete the survey (mean completion time = 10′ 50′′, *SD* = 5′ 36′′). In order to be eligible to participate, participants had to be located in the United States and have a lifetime HIT approval rate of 95% or higher. Three participants who failed to provide any responses to the news articles were excluded from the sample, as no other measures of reactions to discovery were included. The same inclusion criteria were used as in Study 1 and resulted in a slightly greater final sample size (*N* = 505) than the target sample size (*N* = 500).

#### Procedure

After providing informed consent, participants were randomly assigned to read either a news article about the scientific discovery of microbial life on Mars, or one about scientists creating a synthetic cell on Earth. The articles were selected from the New York Times and information regarding the source and date of publication of each article was removed. Participants were randomly assigned to condition (*N* = 256 in the Mars Meteorite condition, *N* = 249 in the Synthetic Life condition). After reading the assigned article, participants were asked to provide a description of their thoughts, feelings, and reactions to the discovery they had just read about in an open response format. The prompts read, “Please take a moment to share your reactions to this scientific discovery. Please provide as much detail as you can and please try to write at least a few sentences describing what YOUR thoughts, feelings, and responses are.” As in Study 1, participants then completed the TIPI [Openness: α = 0.49, Conscientiousness: α = 0.66, Extraversion: α = 0.77, Agreeableness: α = 0.47; Emotional Stability: α = 0.76, and the Disease Avoidance subscale of the Fundamental Social Motives Inventory (α = 0.90)], and demographic questions including items assessing age, gender, ethnicity, country of residence, country of birth, income, education, and political orientation. Due to experimenter error, the PANAS and Likert-scale measures of reward/risk orientation were omitted from Study 2 (see osf.io/mgkau). Study 2 was approved by the institutional review board at Arizona State University.

### Results

Linguistic Inquiry and Word Count text analyses were followed by paired-samples *t*-tests comparing the proportions of words reflecting positive vs. negative affect within each experimental condition (Mars vs. Earth article). Participants who read about microbial life on Mars used more words reflecting positive (*M* = 4.69, *SD* = 7.24) than negative affect (*M* = 0.52, *SD* = 1.10), *t*(255) = 9.06, *p* < 0.001, *d* = 0.80 (see **Figure 1**), and used more words reflecting reward (*M* = 1.33, *SD* = 1.70) than risk (*M* = 0.26, *SD* = 0.69), *t*(255) = 9.66, *p* < 0.001, *d* = 0.83. Participants who read about the synthetic cell used more words reflecting positive (*M* = 5.10, *SD* = 4.07) than negative affect (*M* = 2.01, *SD* = 2.31), *t*(248) = 9.95, *p* < 0.001, *d* = 0.93, and used more words reflecting reward (*M* = 1.88, *SD* = 3.77) than risk (*M* = 1.05, *SD* = 1.47), *t*(248) = 3.28, *p* = 0.001, *d* = 0.29.

A two-way mixed-design ANOVA revealed a marginally significant interaction between the type of article (Martian life vs. synthetic life) and affect (positive vs. negative), *F*(1,503) = 3.73, *p* = 0.05, $\eta_{p}^{2}$ = 0.007, such that reactions of those in the Martian life condition showed a stronger positivity bias than reactions of those in the synthetic life condition. There was no interaction between the type of article and reward vs. risk, *F*(1,503) = 0.76, *p* = 0.38, $\eta_{p}^{2}$ = 0.002.

#### Individual Differences

We did not find particularly strong or consistent correlations between our dependent variables and our individual difference and demographic measures. As for Study 1, here we report only correlations with an absolute value of 0.2 or higher. There was a negative correlation between emotional stability and proportion of words reflecting risk, *r*(254) = -0.209, *p* < 0.001, and between conscientiousness and proportion of words reflecting positive affect, *r*(247) = -0.255, *p* < 0.001. Full correlation matrices, including correlations among dependent variables, can be found at osf.io/mgkau and are also available in the **Appendix**.

### Discussion

Consistent with our preregistered predictions and the results the Pilot and Study 1, we found that people’s responses show more positive vs. negative affect and more orientation to reward vs. risk when confronted with an actual announcement of the discovery of extraterrestrial microbial life. Thus, it appears that this positivity bias is observed not only in cultural products reflecting reactions to such discoveries, or in people’s forecasting of their own and humanity’s reactions, but also in people’s actual reactions to such an announcement. To our knowledge, this is the first empirical test of people’s reactions to an actual announcement of this nature. It is also noteworthy that this positivity bias was more pronounced in response to the discovery of new life of extraterrestrial origin vs. manmade origin, suggesting our findings are not due to a general positivity bias in language or in reactions to the discovery of new life *per se*.

## Mini-Meta-Analysis: Effect Size Comparisons

**FIGURE 2:**
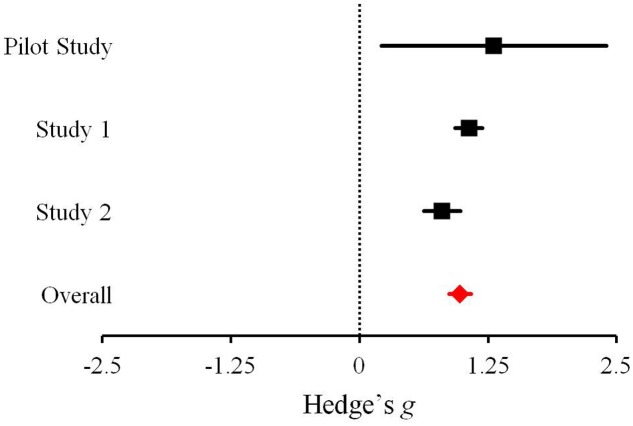
Effect sizes across the three studies examining the difference between the proportion of words reflecting positive vs. negative affect in response to the discovery of extraterrestrial microbial life. Bars represent 95% confidence intervals.

**FIGURE 3:**
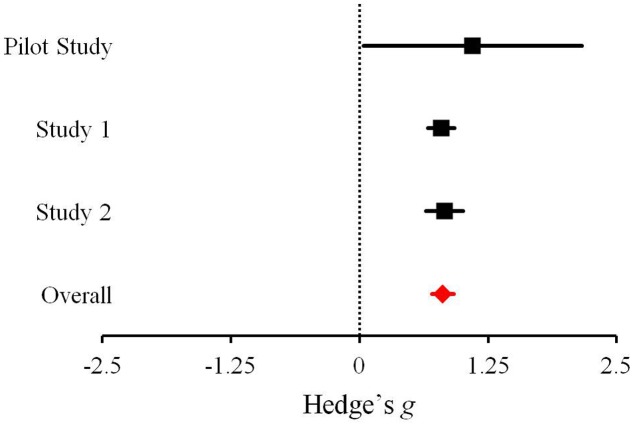
Effect sizes across the three studies examining the difference between the proportion of words reflecting reward vs. risk in response to the discovery of extraterrestrial microbial life. Bars represent 95% confidence intervals.

As our focus was on people’s reactions to announcements regarding the discovery of evidence for extraterrestrial microbial life, we compared the effect sizes of the differences in language used in independent samples across the three studies. Hedge’s *g* effect size estimates, correcting for bias (Hedges, 1981), were calculated for positive vs. negative affect language comparisons of the seven Mars-related articles in the Pilot Study, *g* = 1.31, *p* = 0.019, 95% confidence interval (CI) = [0.213, 2.400], participants’ own predicted reactions in Study 1, *g* = 1.07, *p* < 0.001, 95% CI = [0.932, 1.197], and reactions by those assigned to the Mars meteorite article in Study 2, *g* = 0.80, *p* < 0.001, 95% CI = [0.624, 0.983]. The effect size estimate for reward vs. risk language comparisons in the Pilot Study was *g* = 1.10, *p* = 0.042, 95% CI = [0.039, 2.163]; in Study 1, for participants’ own predicted reactions, *g* = 0.80, *p* < 0.001, 95% CI = [0.667, 0.925]; in Study 2, for those in the Mars meteorite article condition, *g* = 0.83, *p* < 0.001, 95% CI = [0.647, 1.008]. The overall Hedge’s *g* was calculated separately for positive vs. negative affect, *g* = 0.98, *p* < 0.001, 95% CI = [0.870, 1.082], and reward vs. risk language, *g* = 0.81, *p* < 0.001, 95% CI = [0.705, 0.914] (**Figures 2**, **3**, respectively). As defined by Cohen (1992) all of these effect sizes can be considered large.

## General Discussion

In a series of studies, we sought to systematically assess how people may react to the discovery of life that is extraterrestrial in origin. Although this topic has generated a great deal of speculation over the years both within and outside academia, it has received scant empirical attention. Across a pilot study assessing media coverage and two well-powered studies assessing individual reactions, we find fairly consistent evidence that past reactions have been positive, that people believe future reactions will be positive, and that people actually react in a positive fashion to announcements of the discovery of extraterrestrial life. This pattern was observed both when people were asked to forecast their own reactions and those of humanity (Study 1), and was stronger in response to actual announcements of the discovery of novel extraterrestrial life vs. novel man-made forms of life (Study 2). The mini-meta analysis suggests that effects sizes were large and fairly comparable across studies, and that the overall effect sizes for positive vs. negative affect and reward vs. risk orientation in language use were large. Taken together, we believe this work strongly suggests that if we do discover life of non-earthly origin, on the whole, human beings and human societies are likely to respond positively.

We observed one exception to this otherwise consistent pattern in Study 1. On two Likert-scale items intended to assess perceived reward and risk of a hypothetical discovery of extraterrestrial microbial life, participants indicated that they would perceive such a discovery as presenting more potential risks vs. rewards. This may be due to the fact that we assessed this question in a fairly simplistic way using two novel items. However, it may reflect a real difference in people’s spontaneous open-ended responses to such a discovery vs. reactions that may be somewhat more calculated or focused on the dimension of reward vs. risk. Although both questions captured responses in the span of a few minutes, potentially, this opens up a question for future research, namely, whether initial reactions to extraterrestrial life are similar to those after some time has passed. Thus, future research might investigate the stability of such reactions over time. The discrepancy between Likert responses and LIWC results for reward vs. risk in Study 1 may also reflect a limitation of LIWC, as, although LIWC is used to assess underlying feelings and other psychological states based on word use, the relationship between the two is not perfect. That said the two methods despite having little shared variance largely tell a similar story in present studies.

It is also noteworthy that we did not observe much variation in responses as a function of personality traits, disease avoidance, political orientation, or demographic factors such as income or ethnicity. One potential interpretation is that there may be a fair amount of homogeneity in reactions to extraterrestrial life, and that the findings of the current study may be broadly generalizable. However, it is worth noting that our samples were restricted to United States respondents, and, given the fact that Americans differ from many other populations on a slew of psychological tendencies (Henrich et al., 2010), we suggest caution in generalizing the present findings beyond the United States. Thus, we hope to eventually replicate this work cross-culturally in order to assess the degree to which our findings generalize and to explore the possibility that how people react to extraterrestrial life may vary as a function of cultural differences (i.e., differences in values, or socio-ecological conditions). Future studies could also explore whether reactions can be predicted by other individual difference measures related to attitudes toward science in general, such as attitude toward paranormal beliefs or conspiracy mentality. Additionally, religiousness, or particular religious beliefs, may affect how people respond to the discovery of extraterrestrial life. We did not assess these traits in the present work, although we think it may be informative to do so in the future as these may potentially provide boundary conditions for the effects observed in the present research.

We also observed that people’s forecasts regarding their own reactions to a hypothetical discovery of extraterrestrial microbial life showed a stronger positivity bias than their forecasts regarding humanity’s reactions to such a discovery. This may reflect illusory superiority (Brown, 1986), although why positive reactions to alien life would be seen as a desirable trait is a question for future research. However, this discrepancy might in part reflect why some past speculation regarding societal reactions to this type of discovery have been fairly pessimistic. However, it is worth noting that the difference in positivity bias did not reflect a difference in the overall direction of the bias, merely its strength.

In addition, we focused our work on reactions to microbial life, but it may well be that the discovery of intelligent extraterrestrial life might lead to very different types of reactions, as intelligent beings provide different threats and opportunities than microbes. To what extent results might be similar or different is an empirical question, albeit one which may be somewhat difficult to test short of an extremely convincing and immersive psycho-drama in which access to outside information is severely curtailed. Such work would present many challenges, especially in the context of an online study or a laboratory experiment. In addition, given that the likelihood of our species making contact with, or finding convincing proof of, intelligent extraterrestrial life is far smaller than the likelihood that we encounter evidence of current or extinct extraterrestrial microbial life, it may be wiser to focus our resources on preparing for the potential societal ramifications of the latter. That said, recent polls suggest the majority of Americans, British, and Germans believe that some form of extraterrestrial life exists, and large percentages of Americans believe that not only does intelligent extraterrestrial life exist, but also that it has already visited us (Main, 2016). And yet, in none of these societies have we seen an utter breakdown in social order or panic as a result of these widespread beliefs.

In the Pilot Study, we examined whether reactions in the articles differ for the three events covered, as each event may be linked to different forms of extraterrestrial life with varying degrees of complexity and intelligence. While we found no differences across the events in the proportions of words reflecting positive or negative affect, we did find that the articles about the discovery of Earth-like exoplanets tended to convey more reward than risk, compared to the articles about microbial life on Mars. Although it is unknown what forms of life could potentially inhabit these newly discovered planets, such conditions similar to Earth may suggest life forms more readily associated with benefits for humanity, compared to microbial life for which a dynamic interaction with humanity may be more difficult to imagine. Nevertheless, the Pilot Study was limited in its ability to address the question of whether people would react differently toward various forms of alien life, as it contained just a small sample of media coverage, in which no direct announcements or claims were made of discovering new types of life, and as the results may not generalize to individual reactions. Future research should use more direct, large-scale tests of reactions to different forms of extraterrestrial life.

We also wish to highlight some considerations to be made when using news articles for similar studies in the future. In the Pilot Study, the articles were selected from well-known sources with generally high scientific standards. However, it would be interesting to explore whether other news outlets that have lower standards for scientific reporting, or favor sensationalism, would show the same positivity bias. Another limitation worth noting is that in Study 2 we opted to use real newspaper articles covering scientific discoveries and these articles differed in length (Mars Meteorite article: 1555 words, Synthetic Life article: 1053 words). We did so as this had the benefit of helping us to gauge reactions to a real past announcement of ET life and as it avoiding confounds, biases, and participant suspicion that may have arisen had we generated our own materials. However, it is possible that the difference in positivity bias across these two conditions might have been related to differences in article length, although we are not aware of research suggesting that people respond more positively (vs. negatively) to longer vs. shorter texts, nor are we aware of research suggesting that strength of emotional responses in general should be greater for shorter texts. That said, future researchers who wish to replicate or build upon the present work should attend carefully to the issue regarding the length of experimental stimuli to avoid this potential confound.

Finally, the present work is in many ways a stepping-stone. We know that people appear to respond positively to the discovery of extraterrestrial microbes, but we do not know why. Perhaps such news causes people to take comfort in the fact that we are not alone in the universe. Perhaps it strengthens their worldviews, be they religious or scientific. Perhaps it speaks to their desire for novelty. At present, we do not know the mechanisms by which this effect occurs, and we encourage future researchers to test these and other possibilities.

We began this paper with a question: how will we react when we learn that alien life has been discovered? If our findings provide a reasonable guide, then the answer appears to be that we will take it rather well.

## Data Availability

All materials, raw data, coded data, and preregistered predictions are freely available at the Open Science Framework, and can be accessed at osf.io/mgkau.

## Ethics Statement

This study was carried out in accordance with the recommendations of the Institutional Review Board at Arizona State University with written informed consent from all subjects. All subjects gave written informed consent in accordance with the Declaration of Helsinki. The protocol was approved by the Institutional Review Board at Arizona State University.

## Author Contributions

All authors listed have made a substantial, direct and intellectual contribution to the work, and approved it for publication.

## Conflict of Interest Statement

The authors declare that the research was conducted in the absence of any commercial or financial relationships that could be construed as a potential conflict of interest.

## Footnotes

1 The Drake Equation estimates the number of civilizations in our galaxy we could potentially communicate with based on a set of factors including the rate of formation of stars, the fraction of stars with planets capable of hosting life, the fraction of planets on which intelligent life might be expected to emerge, the fraction of planets on which civilizations capable of interstellar transmission might exist, and the length of time for which such civilizations might be detectable (Burchell, 2006).

2 In each study a small number of participants whose data was included in the final analyses did not receive payment for their participation due to failure to submit the HIT on MTurk immediately after completion of their participation. We regret that this occurred. We note that we have no way of reliably identifying these participants from the data we collected, so that it is not possible to exclude their data from analysis.

## Appendix

**Table A1:** Zero-order correlations between demographic items, the five-factor personality traits, disease avoidance motive, and reactions to discovery of extraterrestrial life (Study 1).

	Own reactions								Humanity’s reactions			
	LIWC positive affect	LIWC negative affect	PANAS positive	PANAS negative	LIWC reward	LIWC risk	Likert reward	Likert risk	LIWC positive affect	LIWC negative affect	LIWC reward	LIWC risk
Age	-0.061	-0.025	-0.008	**-0.131^∗∗^**	-0.055	**0.108^∗^**	-0.008	0.021	0.009	0.002	-0.084	-0.039
Gender	-0.022	-0.031	-0.072	-0.003	**0.091^∗^**	0.018	0.014	**0.101^∗^**	0.005	0.004	-0.019	0.000
Household income	-0.021	**0.138^∗∗^**	0.023	0.002	0.057	0.047	0.024	-0.013	-0.022	0.022	0.064	0.019
Education level	-0.030	-0.021	0.011	0.001	-0.042	0.031	-0.056	-0.042	0.047	-0.002	0.072	0.045
Political orientation	**-0.135^∗∗^**	-0.021	**-0.113^∗^**	0.015	-0.086	0.011	**0.125^∗∗^**	0.053	**-0.093^∗^**	0.003	-0.047	-0.009
Extraversion	-0.015	0.026	0.072	0.067	-0.032	0.031	0.013	0.013	-0.061	0.048	0.044	-0.016
Agreeableness	**-0.171^∗∗^**	-0.064	**0.145^∗∗^**	-0.009	-0.079	-0.006	**-0.148^∗∗^**	-0.001	-0.012	**-0.104^∗^**	0.051	-0.048
Conscientiousness	-0.074	-0.026	0.059	0.008	-0.024	-0.032	-0.055	-0.065	0.002	0.029	0.013	0.002
Emotional stability	-0.061	-0.072	**0.096^∗^**	0.053	0.014	-0.018	-0.054	-0.067	0.006	-0.005	-0.027	-0.050
Openness	0.039	0.027	**0.142^∗∗^**	**-0.100^∗^**	0.016	-0.010	**-0.181^∗∗^**	-0.038	-0.040	0.035	-0.028	0.007
Disease avoidance	0.005	-0.003	-0.044	-0.021	-0.014	-0.052	0.061	**0.214^∗∗^**	-0.061	-0.041	-0.033	-0.031

^∗∗^*Correlation is significant at the 0.01 level (2-tailed). ^∗^Correlation is significant at the 0.05 level (2-tailed)*.

**Table A2:** Zero-order correlations between measures of reactions to discovery of extraterrestrial life (Study 1).

		Own reactions								Humanity’s reactions			
		1	2	3	4	5	6	7	8	9	10	11	12
**Own**	(1) LIWC positive affect												
**reactions**	(2) LIWC negative affect	0.001											
**(1–8)**	(3) PANAS positive	0.056	0.041										
	(4) PANAS negative	0.029	0.087	0.061									
	(5) LIWC reward	**0.385^∗∗^**	-0.066	**0.093^∗^**	0.007								
	(6) LIWC risk	**-0.124^∗∗^**	**0.387^∗∗^**	-0.038	0.042	-0.026							
	(7) Likert reward	-0.019	0.001	**-0.521^∗∗^**	0.053	-0.075	-0.019						
	(8) Likert risk	-0.085	**0.175^∗∗^**	-0.004	**0.167^∗∗^**	-0.057	**0.107^∗^**	-0.026					
**Humanity’s**	(9) LIWC positive affect	**0.195^∗∗^**	-0.055	0.010	0.081	**0.130^∗∗^**	-0.015	0.001	**-0.181^∗∗^**				
**reactions**	(10) LIWC negative affect	0.039	**0.135^∗∗^**	**0.122^∗∗^**	0.077	0.046	0.044	0.026	**0.099^∗^**	**-0.230^∗∗^**			
**(9–12)**	(11) LIWC reward	0.022	-0.066	-0.025	0.063	**0.145^∗∗^**	-0.043	-0.018	-0.070	**0.427^∗∗^**	**-0.121^∗∗^**		
	(12) LIWC risk	-0.053	0.018	0.048	**0.126^∗∗^**	0.072	**0.139^∗∗^**	0.012	0.052	**-0.107^∗^**	**0.228^∗∗^**	-0.022	

^∗∗^*Correlation is significant at the 0.01 level (2-tailed). ^∗^Correlation is significant at the 0.05 level (2-tailed)*.

**Table A3:** Zero-order correlations between demographic items, the five-factor personality traits, disease avoidance motive, and reactions to announcements of discovering of evidence for life on mars or creating a synthetic cell on earth (Study 2).

	Mars meteorite condition				Earth synthetic life condition			
	Positive affect	Negative affect	Reward	Risk	Positive affect	Negative affect	Reward	Risk
Age	-0.015	-0.037	0.073	-0.031	-0.099	0.013	-0.045	0.107
Gender	-0.049	0.065	0.023	0.011	-0.019	0.018	0.043	0.016
Household Income	-0.072	-0.032	0.014	0.006	0.039	0.019	0.107	0.111
Education level	0.037	-0.018	0.010	0.084	0.104	0.021	**0.146^∗^**	0.008
Political orientation	-0.099	**0.148^∗^**	-0.063	-0.013	**0.139^∗^**	0.002	0.118	0.004
Extraversion	-0.021	-0.040	-0.014	-0.111	0.008	-0.056	0.064	-0.051
Agreeableness	0.008	0.009	0.057	-0.033	**-0.151^∗^**	0.057	-0.105	-0.013
Conscientiousness	-0.031	-0.096	-0.016	-0.107	**-0.255^∗∗^**	0.021	-0.086	0.113
Emotional stability	-0.106	-0.103	-0.048	**-0.209^∗∗^**	-0.069	-0.074	-0.047	-0.017
Openness	-0.076	-0.078	0.054	-0.053	-0.082	0.021	0.023	0.105
Disease avoidance	-0.024	-0.067	-0.019	-0.003	-0.095	0.015	-0.023	0.071

^∗∗^*Correlation is significant at the 0.01 level (2-tailed). ^∗^Correlation is significant at the 0.05 level (2-tailed)*.

**Table A4:** Zero-order correlations between measures of reactions to announcements of discovering evidence for life on mars or creating a synthetic cell on earth (Study 2).

	Mars meteorite condition				Earth synthetic life condition			
	Positive affect	Negative affect	Reward	Risk	Positive affect	Negative affect	Reward	Risk
Positive affect								
Negative affect	-0.029				-0.114			
Reward	0.068	-0.027			**0.675**^∗∗^	-0.028		
Risk	0.030	**0.519^∗∗^**	0.082		-0.119	0.**431**^∗∗^	0.018	

^∗∗^*Correlation is significant at the 0.01 level (2-tailed). ^∗^Correlation is significant at the 0.05 level (2-tailed)*.
